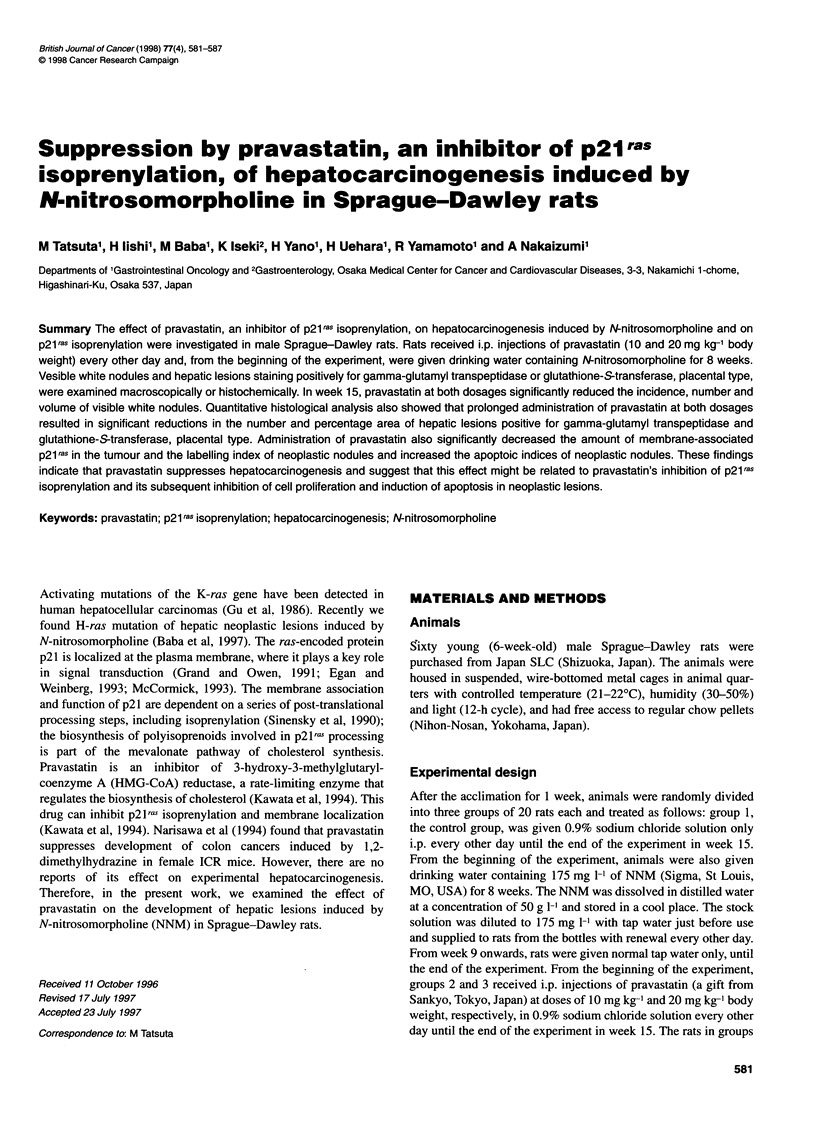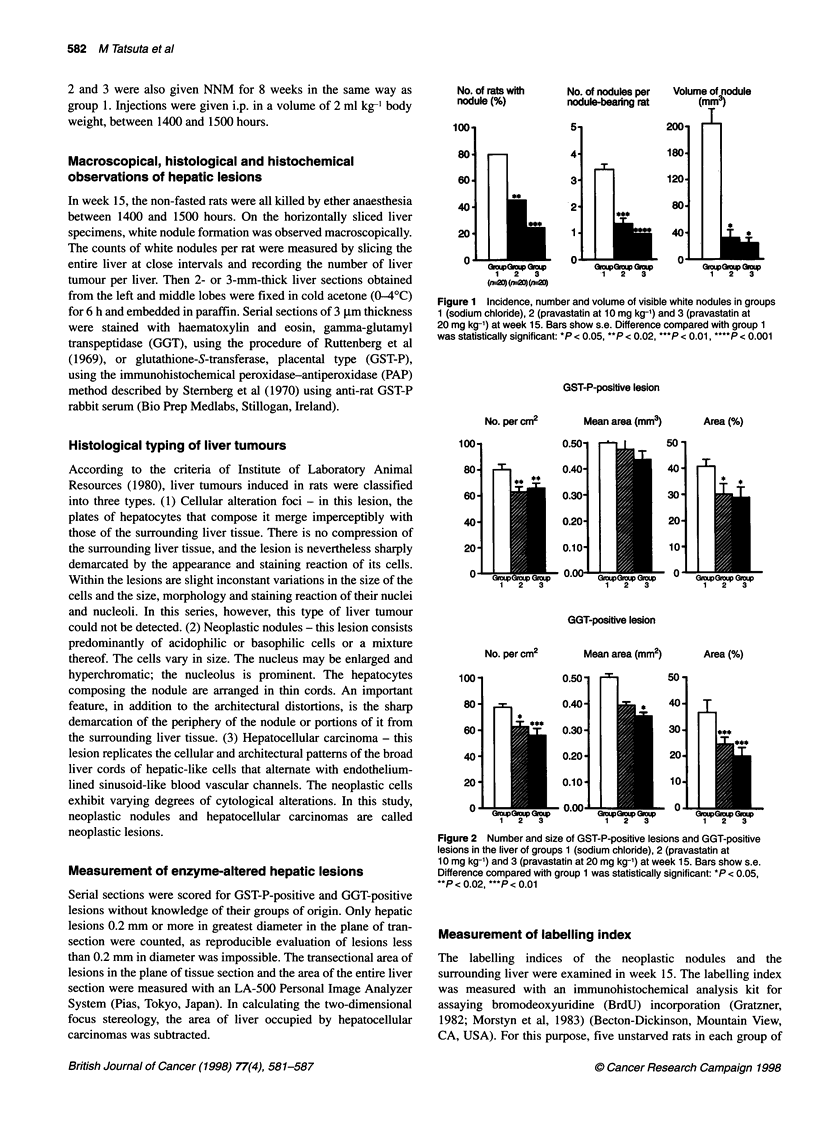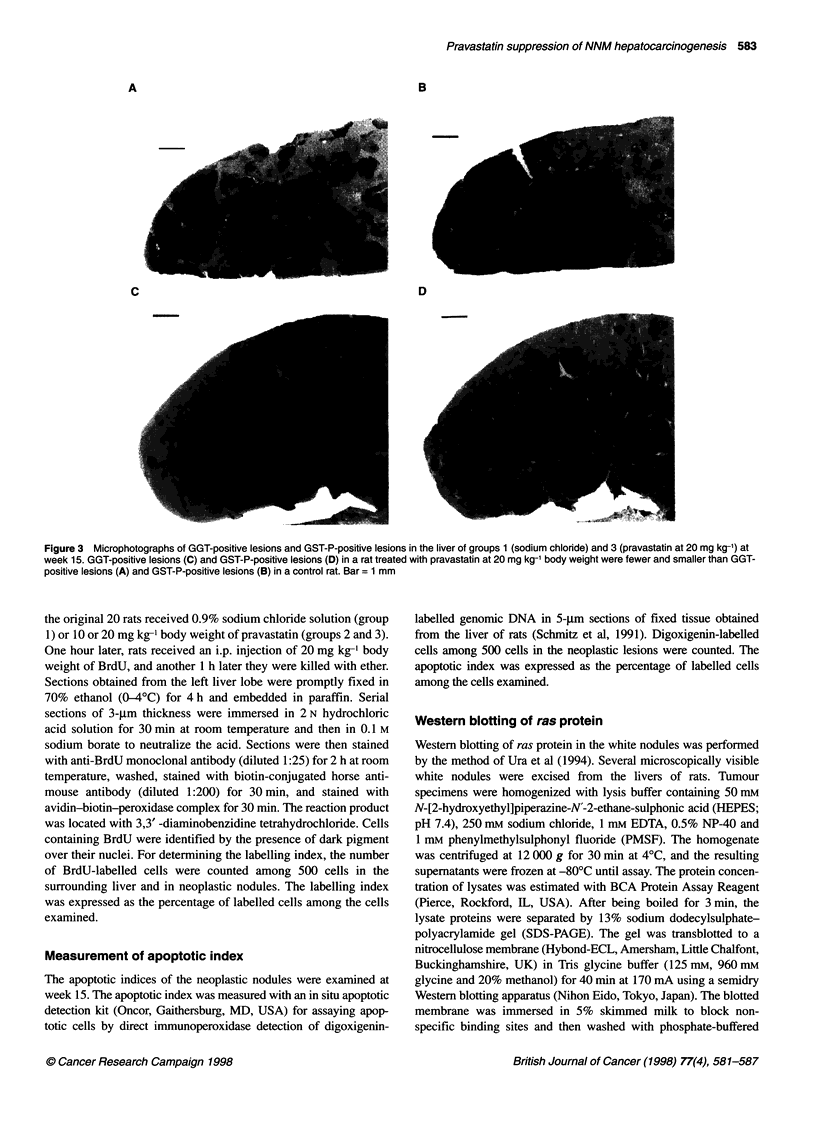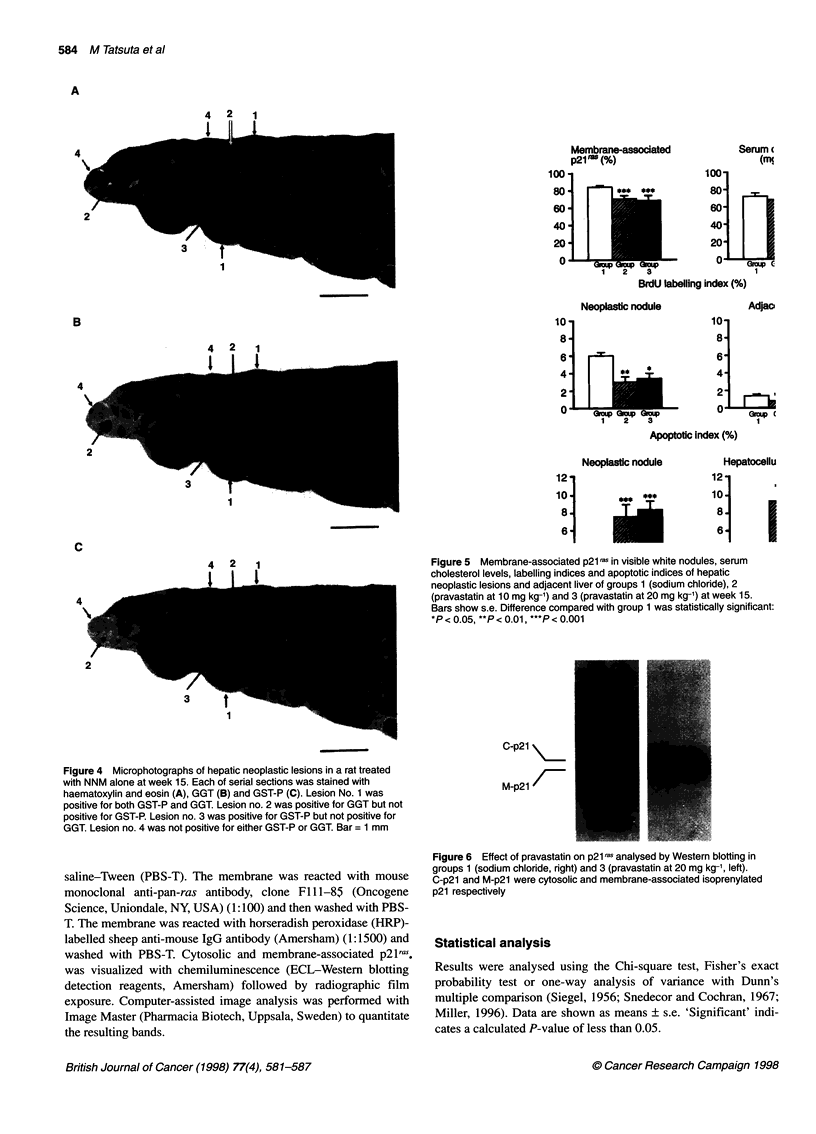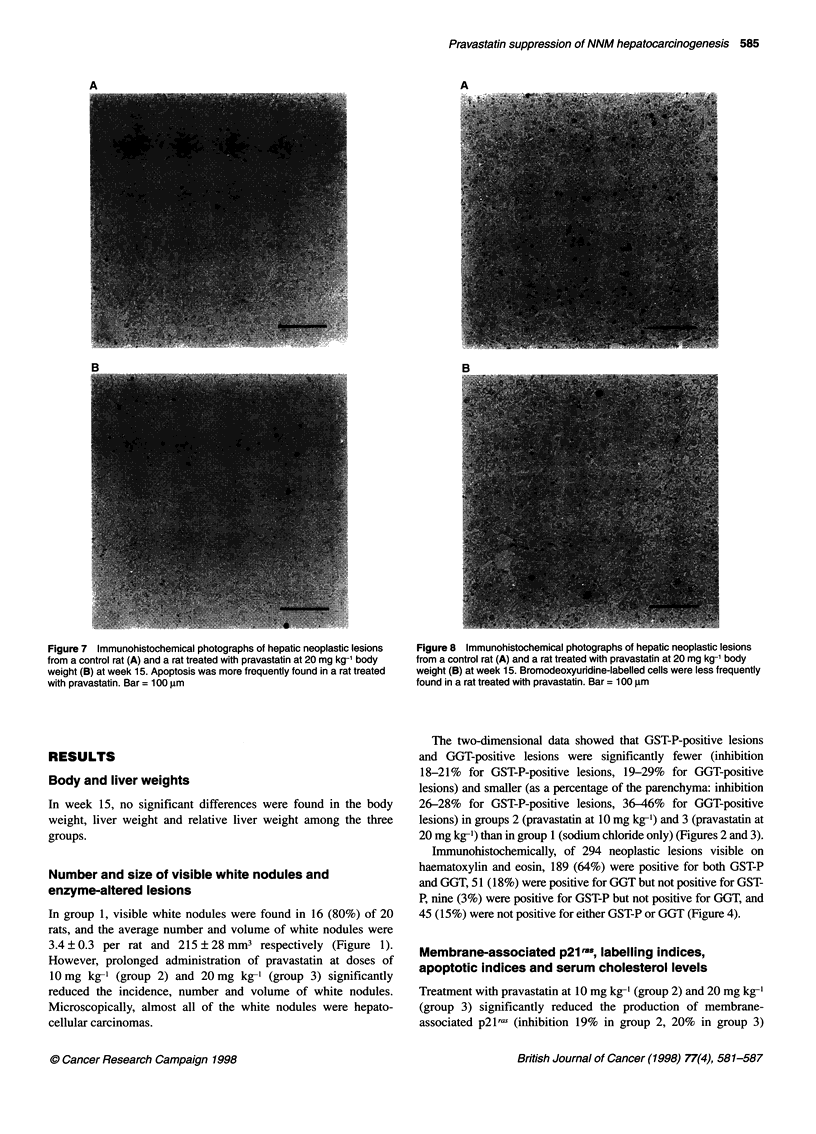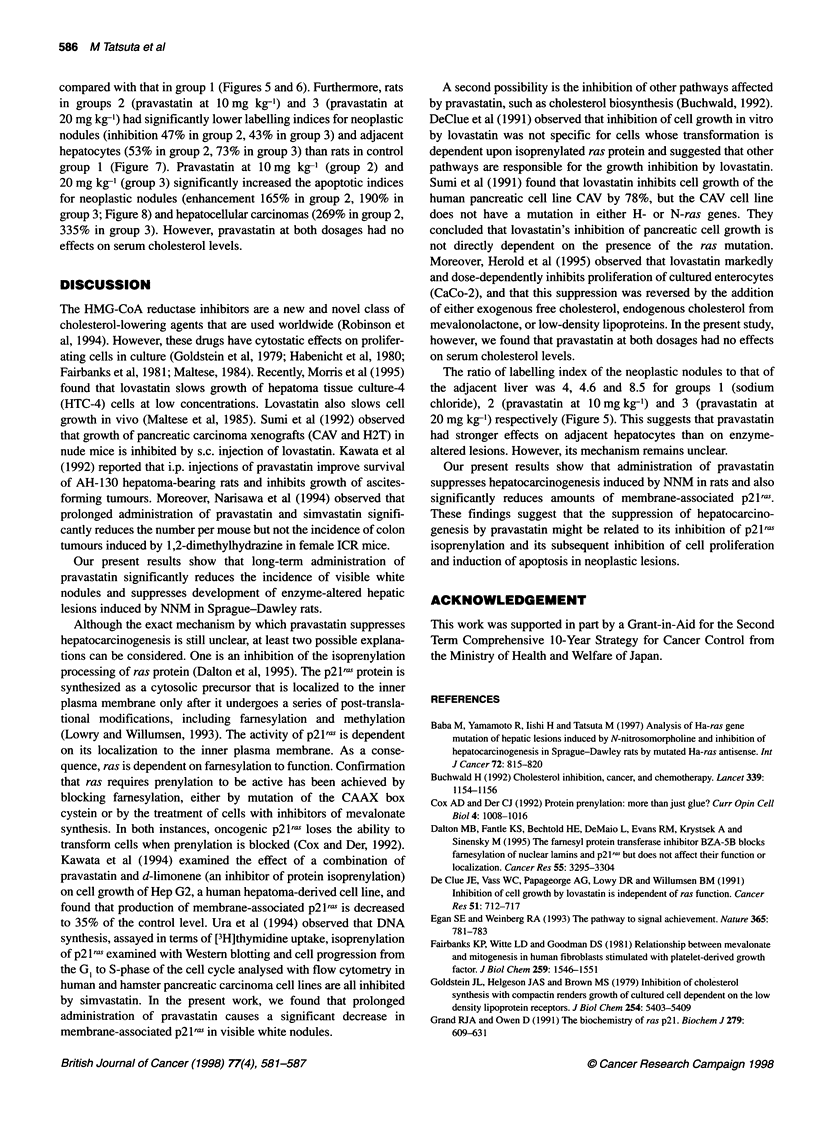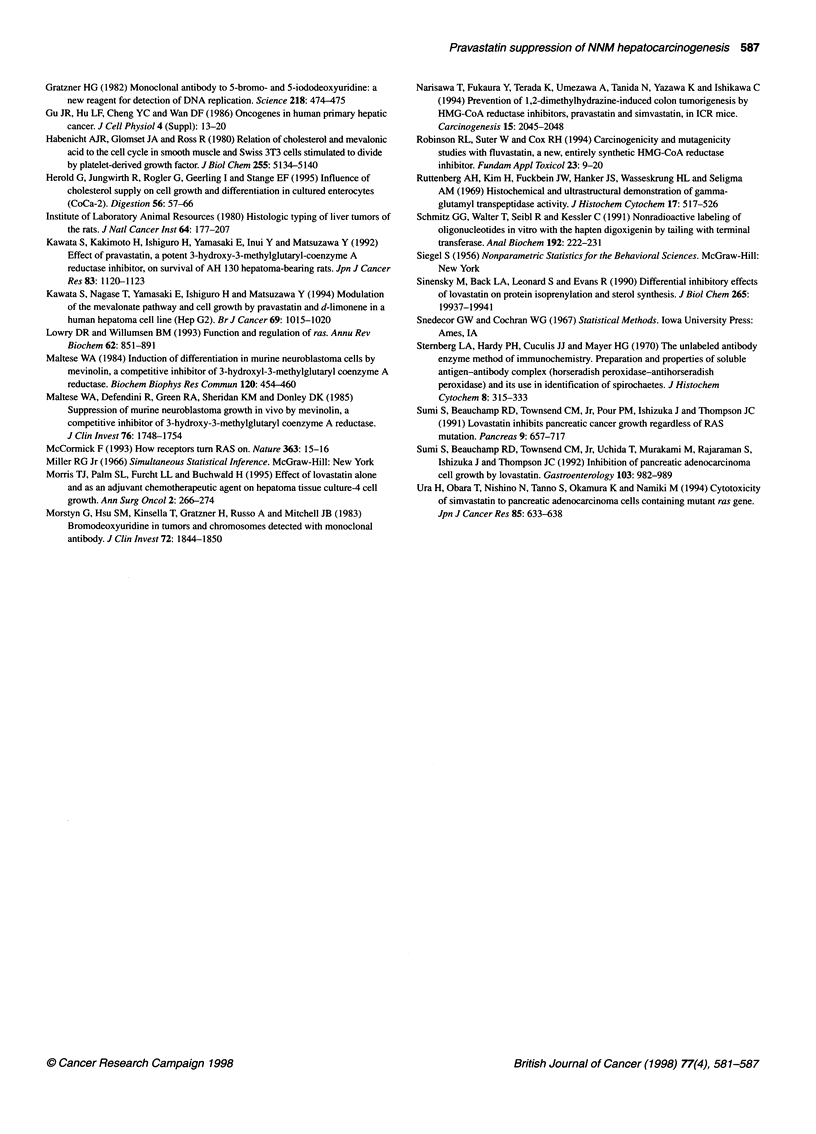# Suppression by pravastatin, an inhibitor of p21ras isoprenylation, of hepatocarcinogenesis induced by N-nitrosomorpholine in Sprague-Dawley rats.

**DOI:** 10.1038/bjc.1998.94

**Published:** 1998-02

**Authors:** M. Tatsuta, H. Iishi, M. Baba, K. Iseki, H. Yano, H. Uehara, R. Yamamoto, A. Nakaizumi

**Affiliations:** Department of Gastrointestinal Oncology, Osaka Medical Center for Cancer and Cardiovascular Diseases, Japan.

## Abstract

**Images:**


British Journal of Cancer (1998) 77(4), 581–587
© 1998 Cancer Research Campaign

### Suppression by pravastatin, an inhibitor of p21$^{ras}$ isoprenylation, of hepatocarcinogenesis induced by *N*-nitrosomorpholine in Sprague–Dawley rats

**M Tatsuta[1], H Iishi[1], M Baba[1], K Iseki[2], H Yano[1], H Uehara[1], R Yamamoto[1] and A Nakaizumi[1]**

Departments of [1]Gastrointestinal Oncology and [2]Gastroenterology, Osaka Medical Center for Cancer and Cardiovascular Diseases, 3-3, Nakamichi 1-chome, Higashinari-Ku, Osaka 537, Japan

**Summary** The effect of pravastatin, an inhibitor of p21$^{ras}$ isoprenylation, on hepatocarcinogenesis induced by *N*-nitrosomorpholine and on p21$^{ras}$ isoprenylation were investigated in male Sprague–Dawley rats. Rats received i.p. injections of pravastatin (10 and 20 mg kg$^{-1}$ body weight) every other day and, from the beginning of the experiment, were given drinking water containing *N*-nitrosomorpholine for 8 weeks. Vesible white nodules and hepatic lesions staining positively for gamma-glutamyl transpeptidase or glutathione-*S*-transferase, placental type, were examined macroscopically or histochemically. In week 15, pravastatin at both dosages significantly reduced the incidence, number and volume of visible white nodules. Quantitative histological analysis also showed that prolonged administration of pravastatin at both dosages resulted in significant reductions in the number and percentage area of hepatic lesions positive for gamma-glutamyl transpeptidase and glutathione-*S*-transferase, placental type. Administration of pravastatin also significantly decreased the amount of membrane-associated p21$^{ras}$ in the tumour and the labelling index of neoplastic nodules and increased the apoptoic indices of neoplastic nodules. These findings indicate that pravastatin suppresses hepatocarcinogenesis and suggest that this effect might be related to pravastatin's inhibition of p21$^{ras}$ isoprenylation and its subsequent inhibition of cell proliferation and induction of apoptosis in neoplastic lesions.

**Keywords:** pravastatin; p21$^{ras}$ isoprenylation; hepatocarcinogenesis; *N*-nitrosomorpholine

Activating mutations of the K-*ras* gene have been detected in human hepatocellular carcinomas (Gu et al, 1986). Recently we found H-*ras* mutation of hepatic neoplastic lesions induced by *N*-nitrosomorpholine (Baba et al, 1997). The *ras*-encoded protein p21 is localized at the plasma membrane, where it plays a key role in signal transduction (Grand and Owen, 1991; Egan and Weinberg, 1993; McCormick, 1993). The membrane association and function of p21 are dependent on a series of post-translational processing steps, including isoprenylation (Sinensky et al, 1990); the biosynthesis of polyisoprenoids involved in p21$^{ras}$ processing is part of the mevalonate pathway of cholesterol synthesis. Pravastatin is an inhibitor of 3-hydroxy-3-methylglutaryl-coenzyme A (HMG-CoA) reductase, a rate-limiting enzyme that regulates the biosynthesis of cholesterol (Kawata et al, 1994). This drug can inhibit p21$^{ras}$ isoprenylation and membrane localization (Kawata et al, 1994). Narisawa et al (1994) found that pravastatin suppresses development of colon cancers induced by 1,2-dimethylhydrazine in female ICR mice. However, there are no reports of its effect on experimental hepatocarcinogenesis. Therefore, in the present work, we examined the effect of pravastatin on the development of hepatic lesions induced by *N*-nitrosomorpholine (NNM) in Sprague–Dawley rats.

*Received 11 October 1996*
*Revised 17 July 1997*
*Accepted 23 July 1997*

*Correspondence to:* M Tatsuta

### MATERIALS AND METHODS

### Animals

Sixty young (6-week-old) male Sprague–Dawley rats were purchased from Japan SLC (Shizuoka, Japan). The animals were housed in suspended, wire-bottomed metal cages in animal quarters with controlled temperature (21–22°C), humidity (30–50%) and light (12-h cycle), and had free access to regular chow pellets (Nihon-Nosan, Yokohama, Japan).

### Experimental design

After the acclimation for 1 week, animals were randomly divided into three groups of 20 rats each and treated as follows: group 1, the control group, was given 0.9% sodium chloride solution only i.p. every other day until the end of the experiment in week 15. From the beginning of the experiment, animals were also given drinking water containing 175 mg l$^{-1}$ of NNM (Sigma, St Louis, MO, USA) for 8 weeks. The NNM was dissolved in distilled water at a concentration of 50 g l$^{-1}$ and stored in a cool place. The stock solution was diluted to 175 mg l$^{-1}$ with tap water just before use and supplied to rats from the bottles with renewal every other day. From week 9 onwards, rats were given normal tap water only, until the end of the experiment. From the beginning of the experiment, groups 2 and 3 received i.p. injections of pravastatin (a gift from Sankyo, Tokyo, Japan) at doses of 10 mg kg$^{-1}$ and 20 mg kg$^{-1}$ body weight, respectively, in 0.9% sodium chloride solution every other day until the end of the experiment in week 15. The rats in groups

2 and 3 were also given NNM for 8 weeks in the same way as group 1. Injections were given i.p. in a volume of 2 ml kg$^{-1}$ body weight, between 1400 and 1500 hours.

### Macroscopical, histological and histochemical observations of hepatic lesions

In week 15, the non-fasted rats were all killed by ether anaesthesia between 1400 and 1500 hours. On the horizontally sliced liver specimens, white nodule formation was observed macroscopically. The counts of white nodules per rat were measured by slicing the entire liver at close intervals and recording the number of liver tumour per liver. Then 2- or 3-mm-thick liver sections obtained from the left and middle lobes were fixed in cold acetone (0–4°C) for 6 h and embedded in paraffin. Serial sections of 3 μm thickness were stained with haematoxylin and eosin, gamma-glutamyl transpeptidase (GGT), using the procedure of Ruttenberg et al (1969), or glutathione-*S*-transferase, placental type (GST-P), using the immunohistochemical peroxidase–antiperoxidase (PAP) method described by Sternberg et al (1970) using anti-rat GST-P rabbit serum (Bio Prep Medlabs, Stillogan, Ireland).

### Histological typing of liver tumours

According to the criteria of Institute of Laboratory Animal Resources (1980), liver tumours induced in rats were classified into three types. (1) Cellular alteration foci – in this lesion, the plates of hepatocytes that compose it merge imperceptibly with those of the surrounding liver tissue. There is no compression of the surrounding liver tissue, and the lesion is nevertheless sharply demarcated by the appearance and staining reaction of its cells. Within the lesions are slight inconstant variations in the size of the cells and the size, morphology and staining reaction of their nuclei and nucleoli. In this series, however, this type of liver tumour could not be detected. (2) Neoplastic nodules – this lesion consists predominantly of acidophilic or basophilic cells or a mixture thereof. The cells vary in size. The nucleus may be enlarged and hyperchromatic; the nucleolus is prominent. The hepatocytes composing the nodule are arranged in thin cords. An important feature, in addition to the architectural distortions, is the sharp demarcation of the periphery of the nodule or portions of it from the surrounding liver tissue. (3) Hepatocellular carcinoma – this lesion replicates the cellular and architectural patterns of the broad liver cords of hepatic-like cells that alternate with endothelium-lined sinusoid-like blood vascular channels. The neoplastic cells exhibit varying degrees of cytological alterations. In this study, neoplastic nodules and hepatocellular carcinomas are called neoplastic lesions.

### Measurement of enzyme-altered hepatic lesions

Serial sections were scored for GST-P-positive and GGT-positive lesions without knowledge of their groups of origin. Only hepatic lesions 0.2 mm or more in greatest diameter in the plane of transection were counted, as reproducible evaluation of lesions less than 0.2 mm in diameter was impossible. The transectional area of lesions in the plane of tissue section and the area of the entire liver section were measured with an LA-500 Personal Image Analyzer System (Pias, Tokyo, Japan). In calculating the two-dimensional focus stereology, the area of liver occupied by hepatocellular carcinomas was subtracted.

**Figure 1** Incidence, number and volume of visible white nodules in groups 1 (sodium chloride), 2 (pravastatin at 10 mg kg$^{-1}$) and 3 (pravastatin at 20 mg kg$^{-1}$) at week 15. Bars show s.e. Difference compared with group 1 was statistically significant: *$P < 0.05$, **$P < 0.02$, ***$P < 0.01$, ****$P < 0.001$

**Figure 2** Number and size of GST-P-positive lesions and GGT-positive lesions in the liver of groups 1 (sodium chloride), 2 (pravastatin at 10 mg kg$^{-1}$) and 3 (pravastatin at 20 mg kg$^{-1}$) at week 15. Bars show s.e. Difference compared with group 1 was statistically significant: *$P < 0.05$, **$P < 0.02$, ***$P < 0.01$

### Measurement of labelling index

The labelling indices of the neoplastic nodules and the surrounding liver were examined in week 15. The labelling index was measured with an immunohistochemical analysis kit for assaying bromodeoxyuridine (BrdU) incorporation (Gratzner, 1982; Morstyn et al, 1983) (Becton-Dickinson, Mountain View, CA, USA). For this purpose, five unstarved rats in each group of

© Cancer Research Campaign 1998

**Figure 3** Microphotographs of GGT-positive lesions and GST-P-positive lesions in the liver of groups 1 (sodium chloride) and 3 (pravastatin at 20 mg kg$^{-1}$) at week 15. GGT-positive lesions (**C**) and GST-P-positive lesions (**D**) in a rat treated with pravastatin at 20 mg kg$^{-1}$ body weight were fewer and smaller than GGT-positive lesions (**A**) and GST-P-positive lesions (**B**) in a control rat. Bar = 1 mm the original 20 rats received 0.9% sodium chloride solution (group 1) or 10 or 20 mg kg$^{-1}$ body weight of pravastatin (groups 2 and 3). One hour later, rats received an i.p. injection of 20 mg kg$^{-1}$ body weight of BrdU, and another 1 h later they were killed with ether. Sections obtained from the left liver lobe were promptly fixed in 70% ethanol (0–4°C) for 4 h and embedded in paraffin. Serial sections of 3-μm thickness were immersed in 2 N hydrochloric acid solution for 30 min at room temperature and then in 0.1 M sodium borate to neutralize the acid. Sections were then stained with anti-BrdU monoclonal antibody (diluted 1:25) for 2 h at room temperature, washed, stained with biotin-conjugated horse anti-mouse antibody (diluted 1:200) for 30 min, and stained with avidin–biotin–peroxidase complex for 30 min. The reaction product was located with 3,3′ -diaminobenzidine tetrahydrochloride. Cells containing BrdU were identified by the presence of dark pigment over their nuclei. For determining the labelling index, the number of BrdU-labelled cells were counted among 500 cells in the surrounding liver and in neoplastic nodules. The labelling index was expressed as the percentage of labelled cells among the cells examined.

### Measurement of apoptotic index

The apoptotic indices of the neoplastic nodules were examined at week 15. The apoptotic index was measured with an in situ apoptotic detection kit (Oncor, Gaithersburg, MD, USA) for assaying apoptotic cells by direct immunoperoxidase detection of digoxigenin-labelled genomic DNA in 5-μm sections of fixed tissue obtained from the liver of rats (Schmitz et al, 1991). Digoxigenin-labelled cells among 500 cells in the neoplastic lesions were counted. The apoptotic index was expressed as the percentage of labelled cells among the cells examined.

### Western blotting of *ras* protein

Western blotting of *ras* protein in the white nodules was performed by the method of Ura et al (1994). Several microscopically visible white nodules were excised from the livers of rats. Tumour specimens were homogenized with lysis buffer containing 50 mM *N*-[2-hydroxyethyl]piperazine-*N*′-2-ethane-sulphonic acid (HEPES; pH 7.4), 250 mM sodium chloride, 1 mM EDTA, 0.5% NP-40 and 1 mM phenylmethylsulphonyl fluoride (PMSF). The homogenate was centrifuged at 12 000 *g* for 30 min at 4°C, and the resulting supernatants were frozen at –80°C until assay. The protein concentration of lysates was estimated with BCA Protein Assay Reagent (Pierce, Rockford, IL, USA). After being boiled for 3 min, the lysate proteins were separated by 13% sodium dodecylsulphate–polyacrylamide gel (SDS-PAGE). The gel was transblotted to a nitrocellulose membrane (Hybond-ECL, Amersham, Little Chalfont, Buckinghamshire, UK) in Tris glycine buffer (125 mM, 960 mM glycine and 20% methanol) for 40 min at 170 mA using a semidry Western blotting apparatus (Nihon Eido, Tokyo, Japan). The blotted membrane was immersed in 5% skimmed milk to block non-specific binding sites and then washed with phosphate-buffered

© Cancer Research Campaign 1998 

Figure 4 Microphotographs of hepatic neoplastic lesions in a rat treated with NNM alone at week 15. Each of serial sections was stained with haematoxylin and eosin (**A**), GGT (**B**) and GST-P (**C**). Lesion No. 1 was positive for both GST-P and GGT. Lesion no. 2 was positive for GGT but not positive for GST-P. Lesion no. 3 was positive for GST-P but not positive for GGT. Lesion no. 4 was not positive for either GST-P or GGT. Bar = 1 mm saline–Tween (PBS-T). The membrane was reacted with mouse monoclonal anti-pan-*ras* antibody, clone F111-85 (Oncogene Science, Uniondale, NY, USA) (1:100) and then washed with PBS-T. The membrane was reacted with horseradish peroxidase (HRP)-labelled sheep anti-mouse IgG antibody (Amersham) (1:1500) and washed with PBS-T. Cytosolic and membrane-associated p21$^{ras}$, was visualized with chemiluminescence (ECL–Western blotting detection reagents, Amersham) followed by radiographic film exposure. Computer-assisted image analysis was performed with Image Master (Pharmacia Biotech, Uppsala, Sweden) to quantitate the resulting bands.

Figure 5 Membrane-associated p21$^{ras}$ in visible white nodules, serum cholesterol levels, labelling indices and apoptotic indices of hepatic neoplastic lesions and adjacent liver of groups 1 (sodium chloride), 2 (pravastatin at 10 mg kg$^{-1}$) and 3 (pravastatin at 20 mg kg$^{-1}$) at week 15. Bars show s.e. Difference compared with group 1 was statistically significant: *$P < 0.05$, **$P < 0.01$, ***$P < 0.001$

Figure 6 Effect of pravastatin on p21$^{ras}$ analysed by Western blotting in groups 1 (sodium chloride, right) and 3 (pravastatin at 20 mg kg$^{-1}$, left). C-p21 and M-p21 were cytosolic and membrane-associated isoprenylated p21 respectively

### Statistical analysis

Results were analysed using the Chi-square test, Fisher's exact probability test or one-way analysis of variance with Dunn's multiple comparison (Siegel, 1956; Snedecor and Cochran, 1967; Miller, 1996). Data are shown as means ± s.e. 'Significant' indicates a calculated *P*-value of less than 0.05.

© Cancer Research Campaign 1998

**Figure 7** Immunohistochemical photographs of hepatic neoplastic lesions from a control rat (**A**) and a rat treated with pravastatin at 20 mg $kg^{-1}$ body weight (**B**) at week 15. Apoptosis was more frequently found in a rat treated with pravastatin. Bar = 100 μm

**Figure 8** Immunohistochemical photographs of hepatic neoplastic lesions from a control rat (**A**) and a rat treated with pravastatin at 20 mg $kg^{-1}$ body weight (**B**) at week 15. Bromodeoxyuridine-labelled cells were less frequently found in a rat treated with pravastatin. Bar = 100 μm

### RESULTS

### Body and liver weights

In week 15, no significant differences were found in the body weight, liver weight and relative liver weight among the three groups.

### Number and size of visible white nodules and enzyme-altered lesions

In group 1, visible white nodules were found in 16 (80%) of 20 rats, and the average number and volume of white nodules were 3.4 ± 0.3 per rat and 215 ± 28 $mm^3$ respectively (Figure 1). However, prolonged administration of pravastatin at doses of 10 mg $kg^{-1}$ (group 2) and 20 mg $kg^{-1}$ (group 3) significantly reduced the incidence, number and volume of white nodules. Microscopically, almost all of the white nodules were hepatocellular carcinomas.

The two-dimensional data showed that GST-P-positive lesions and GGT-positive lesions were significantly fewer (inhibition 18–21% for GST-P-positive lesions, 19–29% for GGT-positive lesions) and smaller (as a percentage of the parenchyma: inhibition 26–28% for GST-P-positive lesions, 36–46% for GGT-positive lesions) in groups 2 (pravastatin at 10 mg $kg^{-1}$) and 3 (pravastatin at 20 mg $kg^{-1}$) than in group 1 (sodium chloride only) (Figures 2 and 3).

Immunohistochemically, of 294 neoplastic lesions visible on haematoxylin and eosin, 189 (64%) were positive for both GST-P and GGT, 51 (18%) were positive for GGT but not positive for GST-P, nine (3%) were positive for GST-P but not positive for GGT, and 45 (15%) were not positive for either GST-P or GGT (Figure 4).

### Membrane-associated p21$^{ras}$, labelling indices, apoptotic indices and serum cholesterol levels

Treatment with pravastatin at 10 mg $kg^{-1}$ (group 2) and 20 mg $kg^{-1}$ (group 3) significantly reduced the production of membrane-associated p21$^{ras}$ (inhibition 19% in group 2, 20% in group 3)

compared with that in group 1 (Figures 5 and 6). Furthermore, rats in groups 2 (pravastatin at 10 mg $kg^{-1}$) and 3 (pravastatin at 20 mg $kg^{-1}$) had significantly lower labelling indices for neoplastic nodules (inhibition 47% in group 2, 43% in group 3) and adjacent hepatocytes (53% in group 2, 73% in group 3) than rats in control group 1 (Figure 7). Pravastatin at 10 mg $kg^{-1}$ (group 2) and 20 mg $kg^{-1}$ (group 3) significantly increased the apoptotic indices for neoplastic nodules (enhancement 165% in group 2, 190% in group 3; Figure 8) and hepatocellular carcinomas (269% in group 2, 335% in group 3). However, pravastatin at both dosages had no effects on serum cholesterol levels.

### DISCUSSION

The HMG-CoA reductase inhibitors are a new and novel class of cholesterol-lowering agents that are used worldwide (Robinson et al, 1994). However, these drugs have cytostatic effects on proliferating cells in culture (Goldstein et al, 1979; Habenicht et al, 1980; Fairbanks et al, 1981; Maltese, 1984). Recently, Morris et al (1995) found that lovastatin slows growth of hepatoma tissue culture-4 (HTC-4) cells at low concentrations. Lovastatin also slows cell growth in vivo (Maltese et al, 1985). Sumi et al (1992) observed that growth of pancreatic carcinoma xenografts (CAV and H2T) in nude mice is inhibited by s.c. injection of lovastatin. Kawata et al (1992) reported that i.p. injections of pravastatin improve survival of AH-130 hepatoma-bearing rats and inhibits growth of ascites-forming tumours. Moreover, Narisawa et al (1994) observed that prolonged administration of pravastatin and simvastatin significantly reduces the number per mouse but not the incidence of colon tumours induced by 1,2-dimethylhydrazine in female ICR mice.

Our present results show that long-term administration of pravastatin significantly reduces the incidence of visible white nodules and suppresses development of enzyme-altered hepatic lesions induced by NNM in Sprague–Dawley rats.

Although the exact mechanism by which pravastatin suppresses hepatocarcinogenesis is still unclear, at least two possible explanations can be considered. One is an inhibition of the isoprenylation processing of *ras* protein (Dalton et al, 1995). The $p21^{ras}$ protein is synthesized as a cytosolic precursor that is localized to the inner plasma membrane only after it undergoes a series of post-translational modifications, including farnesylation and methylation (Lowry and Willumsen, 1993). The activity of $p21^{ras}$ is dependent on its localization to the inner plasma membrane. As a consequence, *ras* is dependent on farnesylation to function. Confirmation that *ras* requires prenylation to be active has been achieved by blocking farnesylation, either by mutation of the CAAX box cystein or by the treatment of cells with inhibitors of mevalonate synthesis. In both instances, oncogenic $p21^{ras}$ loses the ability to transform cells when prenylation is blocked (Cox and Der, 1992). Kawata et al (1994) examined the effect of a combination of pravastatin and *d*-limonene (an inhibitor of protein isoprenylation) on cell growth of Hep G2, a human hepatoma-derived cell line, and found that production of membrane-associated $p21^{ras}$ is decreased to 35% of the control level. Ura et al (1994) observed that DNA synthesis, assayed in terms of $[^3H]$thymidine uptake, isoprenylation of $p21^{ras}$ examined with Western blotting and cell progression from the $G_1$ to S-phase of the cell cycle analysed with flow cytometry in human and hamster pancreatic carcinoma cell lines are all inhibited by simvastatin. In the present work, we found that prolonged administration of pravastatin causes a significant decrease in membrane-associated $p21^{ras}$ in visible white nodules.

A second possibility is the inhibition of other pathways affected by pravastatin, such as cholesterol biosynthesis (Buchwald, 1992). DeClue et al (1991) observed that inhibition of cell growth in vitro by lovastatin was not specific for cells whose transformation is dependent upon isoprenylated *ras* protein and suggested that other pathways are responsible for the growth inhibition by lovastatin. Sumi et al (1991) found that lovastatin inhibits cell growth of the human pancreatic cell line CAV by 78%, but the CAV cell line does not have a mutation in either H- or N-*ras* genes. They concluded that lovastatin's inhibition of pancreatic cell growth is not directly dependent on the presence of the *ras* mutation. Moreover, Herold et al (1995) observed that lovastatin markedly and dose-dependently inhibits proliferation of cultured enterocytes (CaCo-2), and that this suppression was reversed by the addition of either exogenous free cholesterol, endogenous cholesterol from mevalonolactone, or low-density lipoproteins. In the present study, however, we found that pravastatin at both dosages had no effects on serum cholesterol levels.

The ratio of labelling index of the neoplastic nodules to that of the adjacent liver was 4, 4.6 and 8.5 for groups 1 (sodium chloride), 2 (pravastatin at 10 mg $kg^{-1}$) and 3 (pravastatin at 20 mg $kg^{-1}$) respectively (Figure 5). This suggests that pravastatin had stronger effects on adjacent hepatocytes than on enzyme-altered lesions. However, its mechanism remains unclear.

Our present results show that administration of pravastatin suppresses hepatocarcinogenesis induced by NNM in rats and also significantly reduces amounts of membrane-associated $p21^{ras}$. These findings suggest that the suppression of hepatocarcinogenesis by pravastatin might be related to its inhibition of $p21^{ras}$ isoprenylation and its subsequent inhibition of cell proliferation and induction of apoptosis in neoplastic lesions.

### ACKNOWLEDGEMENT

This work was supported in part by a Grant-in-Aid for the Second Term Comprehensive 10-Year Strategy for Cancer Control from the Ministry of Health and Welfare of Japan.

### REFERENCES

Baba M, Yamamoto R, Iishi H and Tatsuta M (1997) Analysis of Ha-*ras* gene mutation of hepatic lesions induced by *N*-nitrosomorpholine and inhibition of hepatocarcinogenesis in Sprague–Dawley rats by mutated Ha-*ras* antisense. *Int J Cancer* **72**: 815–820

Buchwald H (1992) Cholesterol inhibition, cancer, and chemotherapy. *Lancet* **339**: 1154–1156

Cox AD and Der CJ (1992) Protein prenylation: more than just glue? *Curr Opin Cell Biol* **4**: 1008–1016

Dalton MB, Fantle KS, Bechtold HE, DeMaio L, Evans RM, Krystsek A and Sinensky M (1995) The farnesyl protein transferase inhibitor BZA-5B blocks farnesylation of nuclear lamins and $p21^{ras}$ but does not affect their function or localization. *Cancer Res* **55**: 3295–3304

De Clue JE, Vass WC, Papageorge AG, Lowy DR and Willumsen BM (1991) Inhibition of cell growth by lovastatin is independent of *ras* function. *Cancer Res* **51**: 712–717

Egan SE and Weinberg RA (1993) The pathway to signal achievement. *Nature* **365**: 781–783

Fairbanks KP, Witte LD and Goodman DS (1981) Relationship between mevalonate and mitogenesis in human fibroblasts stimulated with platelet-derived growth factor. *J Biol Chem* **259**: 1546–1551

Goldstein JL, Helgeson JAS and Brown MS (1979) Inhibition of cholesterol synthesis with compactin renders growth of cultured cell dependent on the low density lipoprotein receptors. *J Biol Chem* **254**: 5403–5409

Grand RJA and Owen D (1991) The biochemistry of *ras* p21. *Biochem J* **279**: 609–631

© Cancer Research Campaign 1998

Gratzner HG (1982) Monoclonal antibody to 5-bromo- and 5-iododeoxyuridine: a new reagent for detection of DNA replication. *Science* **218**: 474–475

Gu JR, Hu LF, Cheng YC and Wan DF (1986) Oncogenes in human primary hepatic cancer. *J Cell Physiol* **4** (Suppl): 13–20

Habenicht AJR, Glomset JA and Ross R (1980) Relation of cholesterol and mevalonic acid to the cell cycle in smooth muscle and Swiss 3T3 cells stimulated to divide by platelet-derived growth factor. *J Biol Chem* **255**: 5134–5140

Herold G, Jungwirth R, Rogler G, Geerling I and Stange EF (1995) Influence of cholesterol supply on cell growth and differentiation in cultured enterocytes (CoCa-2). *Digestion* **56**: 57–66

Institute of Laboratory Animal Resources (1980) Histologic typing of liver tumors of the rats. *J Natl Cancer Inst* **64**: 177–207

Kawata S, Kakimoto H, Ishiguro H, Yamasaki E, Inui Y and Matsuzawa Y (1992) Effect of pravastatin, a potent 3-hydroxy-3-methylglutaryl-coenzyme A reductase inhibitor, on survival of AH 130 hepatoma-bearing rats. *Jpn J Cancer Res* **83**: 1120–1123

Kawata S, Nagase T, Yamasaki E, Ishiguro H and Matsuzawa Y (1994) Modulation of the mevalonate pathway and cell growth by pravastatin and *d*-limonene in a human hepatoma cell line (Hep G2). *Br J Cancer* **69**: 1015–1020

Lowry DR and Willumsen BM (1993) Function and regulation of *ras*. *Annu Rev Biochem* **62**: 851–891

Maltese WA (1984) Induction of differentiation in murine neuroblastoma cells by mevinolin, a competitive inhibitor of 3-hydroxyl-3-methylglutaryl coenzyme A reductase. *Biochem Biophys Res Commun* **120**: 454–460

Maltese WA, Defendini R, Green RA, Sheridan KM and Donley DK (1985) Suppression of murine neuroblastoma growth in vivo by mevinolin, a competitive inhibitor of 3-hydroxy-3-methylglutaryl coenzyme A reductase. *J Clin Invest* **76**: 1748–1754

McCormick F (1993) How receptors turn RAS on. *Nature* **363**: 15–16

Miller RG Jr (1966) *Simultaneous Statistical Inference*. McGraw-Hill: New York

Morris TJ, Palm SL, Furcht LL and Buchwald H (1995) Effect of lovastatin alone and as an adjuvant chemotherapeutic agent on hepatoma tissue culture-4 cell growth. *Ann Surg Oncol* **2**: 266–274

Morstyn G, Hsu SM, Kinsella T, Gratzner H, Russo A and Mitchell JB (1983) Bromodeoxyuridine in tumors and chromosomes detected with monoclonal antibody. *J Clin Invest* **72**: 1844–1850

Narisawa T, Fukaura Y, Terada K, Umezawa A, Tanida N, Yazawa K and Ishikawa C (1994) Prevention of 1,2-dimethylhydrazine-induced colon tumorigenesis by HMG-CoA reductase inhibitors, pravastatin and simvastatin, in ICR mice. *Carcinogenesis* **15**: 2045–2048

Robinson RL, Suter W and Cox RH (1994) Carcinogenicity and mutagenicity studies with fluvastatin, a new, entirely synthetic HMG-CoA reductase inhibitor. *Fundam Appl Toxicol* **23**: 9–20

Ruttenberg AH, Kim H, Fuckbein JW, Hanker JS, Wasseskrung HL and Seligma AM (1969) Histochemical and ultrastructural demonstration of gamma-glutamyl transpeptidase activity. *J Histochem Cytochem* **17**: 517–526

Schmitz GG, Walter T, Seibl R and Kessler C (1991) Nonradioactive labeling of oligonucleotides in vitro with the hapten digoxigenin by tailing with terminal transferase. *Anal Biochem* **192**: 222–231

Siegel S (1956) *Nonparametric Statistics for the Behavioral Sciences*. McGraw-Hill: New York

Sinensky M, Back LA, Leonard S and Evans R (1990) Differential inhibitory effects of lovastatin on protein isoprenylation and sterol synthesis. *J Biol Chem* **265**: 19937–19941

Snedecor GW and Cochran WG (1967) *Statistical Methods*. Iowa University Press: Ames, IA

Sternberg LA, Hardy PH, Cuculis JJ and Mayer HG (1970) The unlabeled antibody enzyme method of immunochemistry. Preparation and properties of soluble antigen–antibody complex (horseradish peroxidase–antihorseradish peroxidase) and its use in identification of spirochaetes. *J Histochem Cytochem* **8**: 315–333

Sumi S, Beauchamp RD, Townsend CM, Jr, Pour PM, Ishizuka J and Thompson JC (1991) Lovastatin inhibits pancreatic cancer growth regardless of RAS mutation. *Pancreas* **9**: 657–717

Sumi S, Beauchamp RD, Townsend CM, Jr, Uchida T, Murakami M, Rajaraman S, Ishizuka J and Thompson JC (1992) Inhibition of pancreatic adenocarcinoma cell growth by lovastatin. *Gastroenterology* **103**: 982–989

Ura H, Obara T, Nishino N, Tanno S, Okamura K and Namiki M (1994) Cytotoxicity of simvastatin to pancreatic adenocarcinoma cells containing mutant *ras* gene. *Jpn J Cancer Res* **85**: 633–638

© Cancer Research Campaign 1998